# Cannabis and Kratom online information in Thailand: Facebook trends 2015–2016

**DOI:** 10.1186/s13011-018-0155-4

**Published:** 2018-05-09

**Authors:** Kanittha Thaikla, Kanokporn Pinyopornpanish, Wichuda Jiraporncharoen, Chaisiri Angkurawaranon

**Affiliations:** 1 Research Institute for Health Sciences, Chiang Mai University, Chiang Mai, 50200 Thailand; 2 Department of Family Medicine, Faculty of Medicine, Chiang Mai University, Chiang Mai, 50200 Thailand

**Keywords:** Thailand, Internet, Facebook, Psychoactive drug, Kratom, Cannabis, Marijuana

## Abstract

**Background:**

Our study aims to evaluate the trends in online information about cannabis and kratom on Facebook in Thailand, where there is current discussion regarding legalizing these drugs.

**Methods:**

Between April and November 2015, reviewers searched for cannabis and kratom Facebook pages in the Thai language via the common search engines. Content analysis was performed and the contents of each page were categorized by the tone of the post (positive, negative or neutral). Then, a one-year follow-up search was conducted to compare the contents.

**Results:**

Twelve Facebook pages each were initially identified for cannabis and for kratom. Follower numbers were higher for cannabis pages. Kratom pages were less active but were open for a longer time. Posts with positive tones and neutral tones were found for both drugs, but none had negative tones. Other drugs were mentioned on the cannabis pages, but they were different from those mentioned on the kratom pages. Issues regarding drug legalization were found on the cannabis pages but not on the kratom pages during the searching period. One year later, the tone of the posts was in the same direction, but the page activity had increased.

**Conclusions:**

The information currently available on the sampled Facebook pages was positive towards the use of cannabis and kratom. No information about harm from these drugs was found through our search.

## Background

Cannabis (marijuana) and kratom (*Mitragyna speciosa*) have been reported to have increasing worldwide use [7]. Studies have shown many negative impacts on health with excessive use of these substances. [18]. Cannabis use is related to many psychological conditions, cardiovascular events, respiratory tract problems and cognitive impairment [10, 28], while kratom could be associated with stimulant effects and opioid-like side effects. Long-term and high-dose usage of kratom has been associated with weight loss, hyperpigmentation, tremor, insomnia, fatigue, poor concentration and possibly seizures [12, 21, 24]. Due to the potential harm, together with the possibility of misuse by users, the Thai Narcotics Control Division has categorized these plants as illicit drug category V, which is illegal to produce, dispose, import, export or possess [17].

These two drugs have been reported to have various medicinal benefits. Cannabis has been used to decrease nausea, vomiting, and spasticity as well as in pain control and appetite improvement [8]. Kratom was also reported to have positive effects, especially anti-inflammatory, cough-reducing, anti-diarrheal and pain relief effect [12]. Many organizations or groups of people who advocate for legalization use the potential medical benefits of these drugs to drive law changes. However, one study found that cannabis that is legally used for medical purposes is also more likely to be recreationally used by the general population [19]. Thailand is still in discussion regarding legalizing these drugs. As they are still illegal drugs, people are influenced to obtain them from routes outside of government regulation. The online market is one of those routes.

Over the past decade, the internet has become a major location where people communicate and search for information, including that regarding drugs. In Thailand and other countries, illicit drugs and illegal prescriptions drugs are available online with a wide variety of information [4, 11, 15, 20, 22, 23, 27]. The commonly controlled psychoactive substances that have been investigated for online information in prior studies were opiates and cannabis. In contrast, kratom, to the best of our knowledge, has not been studied for online content. This plant is typically used only in a small part of the world, and Thailand is one of the most popular market places [13]. Kratom is currently also available in the European online drug market and is a current topic for legal status debate in the United State. [9, 22, 25]

In recent years, the Thai government has been considering changing the drug schedule for cannabis and kratom. This study aims to evaluate the trend of online information on Facebook pages devoted to cannabis and kratom (the most popular and easily accessible social network site among Thai users) [5] by categorizing their content based on positive, negative, and neutral tones, determining page activities and noting what other drugs are mentioned in conjunction with cannabis or kratom. These findings of this content analysis may help provide indicators of whether cannabis- and kratom-related content posted on Facebook is potentially harmful to social network users. The findings may also help contextualize the current legal uncertainty of cannabis and kratom in Thailand.

## Methods

Surveys of content were conducted twice, 1 year apart. The first round of the survey was conducted between April and November 2015. Reviewers manually searched websites daily by entering keywords for cannabis and kratom in the Thai language via the common search engines: Google, Yahoo, and Ask Bing. New keywords obtained during the search process were then used for extended searches. All the keywords are described in Table 1. We additionally conducted a specific search by using “site: Facebook.com” (e.g., กระท่อม site: Facebook.com) to access more pages directly from Facebook via their search engine. We observed the first 300 websites listed from each search, and only Facebook pages were collected for further analysis.

**Table 1:** Keywords

Drugs	Keywords (Thai Language)
Cannabis (กัญชา)	กัญชา, กัญชาไทย ขายกัญชาออนไลน์, เขียว กัญชา, บ้องกัญชา, กัญชา ยาเสพติด, ไทยสติ๊ก, กัญชา ไทยสติ๊ก
	กัญชา หญ้ายิ้ม, ดูดกัญชา, สูบกัญชา, เป็ดน้อย กัญชา, ยาเส้น กัญชา, เสพกัญชา
	สายเขียว, ขายกัญชา, กัญชาอัดแท่งขายเมล็ดกัญชา. กระตุกบ้องกัญชา, บุหรี่ยัดไส้กัญชา,
	คนกัญชา, กัญชาสายเขียว, กัญชาเดินดง, กัญชา ขาย แจก, กัญชา ขาย ดูด, ยาหม้อ กัญชา, กัญชา ด้ายแดง, กัญชา ใบไม้เมา, ปุ๊นกัญชา สายเขียว, สายเขียว กัญชา, กัญชาผสมน้ำผึ้ง, ขายเนื้อ กัญชา, กัญชาขายยังไง
Kratom (กระท่อม)	กระท่อม, น้ำท่อม, 4*100 ภาคใต้, 4*100 น้ำท่อม, ชนมือ ใบกระท่อม, ใบไม้ยัน,กระท่อม ยัน, ใบไม้เชือน, เอแดง ใบกระท่อม, ใบไม้ขม, กินท่อม ขายน้ำต้มใบกระท่อม,สายเชือน,ท่อมชาเขียว,ชากระท่อม, กระท่อม น้ำต้มใบกระท่อม, ขายใบมัด ใบโล,ใบกระท่อมก้านแดง, เมาน้ำท่อม, ขายใบกระท่อม, กินน้ำท่อม

Thai-language keywords used for searching via the common search engines (Google, Yahoo, and Ask Bing)

We recorded the names of Facebook pages and page URL addresses in order to track the pages. The number of followers was also recorded as a proxy for the popularity of the page. Content analysis of posts was done by a team of reviewers (KP, WJ, CA). Two independent authors (KP and WJ) individually categorized the first 50 posts. Independently, the two reviewers agreed on 88% of the post. The interrater agreement (kappa) for the first 50 posts was 0.72 (appendix 1). They then collaborated to reach conclusions for the first 50 posts, and then discussed with CA if there was any discrepancy. Afterward, the posts were categorized independently by KP and later reviewed by one of the other two reviewers. The tones of the content were analysed related to the drug and divided into three categories which were positive tone, negative tone and neutral tone. This categorization was adapted from a prior study on marijuana-related traffic on Twitter [27]. A positive tone denoted the text and/or pictures that implied a notably positive attitude towards the use of the drugs. Pictures of smoking people, tools to be used with the drugs, ready-to-use drugs in containers and advertising or marketing of drug-related products were also included. Any content against the use of the drugs was categorized as a negative tone. Neutral tone included the contents that were unlikely to convince people to think positively or negatively about the use of the drugs, for example, a general greeting by an administrator, a picture of the plant or leaves without words, news about laws or research findings without an opinion, and any unrelated topic. Posts about changes in profile picture or cover picture were categorized as neutral. The rate of posting was evaluated by the duration between the last post and the tenth post from the last. A longer period showed less activity on the page. The tones of the posts were obtained and analysed from these last ten posts from each page. The names of other drugs that were suggested to be used with cannabis or kratom in pages were collected to estimate the drugs recommended by the page administrators..

The one-year follow-up search was conducted in October 2016 for 1 week using the URL pages that had been used in 2015. Additionally, new searches were conducted using the 2015 keywords. This follow-up was done in order to estimate the activity and availability of the 2015 pages and to evaluate the content posted on the 2016 pages using the same analysis theme.

## Results

Initially, twenty-four total pages were identified, twelve for kratom and twelve for cannabis. These pages had varying numbers of followers. The overall number of followers for cannabis was higher. Posts with positive tones and neutral tones were found for both drugs, but none had negative tones. The results were similar in both periods of searching, 1 year apart. Although there were many posts with a neutral tone about the King who passed away and the country’s great loss during the follow-up period, it seemed that the posts in October 2016 were more likely to have a positive tone about the drugs. Examples of the posts and the categorizations of positive tone are demonstrated in Table 2.

**Table 2:** Illustration of Posts Categorized by tones and topic of interest

Topic	Cannabis	Kratom
Posts with Positive tone		
Persuasive Greeting with/ without Picture	*“Good morning! Have you smoked weed today?”*	*“Admin wants to eat kratom. What about you guys?”*
	*“May 31 is World No Tobacco Day. Cannabis smoking is better.”*	*“Hey my friend, it’s morning! Do you want one pot of this? Ha ha!” (with kratom tea picture)*
	*“I woke up at 3 AM so I used once (This one is portable with the size of your palm).*	*“I was away for long. I’m back with Kratom today!!” (with picture of Kratom leaves and the bottle of cough suppressant)*
		*“What a wonderful wedding ceremony. They serve Kratom tea. Awsome!”*
Mention of Properties/ Benefits	*“Blue Dream is one of the best-seller cannabis in the US. With its anti-depressive and analgesic effect and the berry-like taste, no doubt that it becomes more and more popular.”*	*“If you are not kratom users, you would not know the benefits of it. I tried to find the pros and cons of this drug and found mostly pros. For example, knowing more people, sharing experience, having responsibility, knowing how to manage finances or supply, never causing trouble to other people (better than alcohol). There are some cons, for example, learning impairment (but in my experience my friends have good grades and I want everyone to be like this)…”*
		*“… The medical properties are anti-diarrheal and some would say it could treat diabetes.”*
Opinion	*“Cannabis cures everything.”*	*“It’s friendship. Even we are in different age or different socioeconomic status, when we sit together, we all allies. When we get drunk, we never fight any one. The thing that happens is only friendship.”*
	*“Cannabis kills no one.”*	
	*“Good for your mind. Everyone likes it.”*	
	*“Once you smoke, even you are feeling so sad, you will always have a smile.”*	
	*“Cannabis has never lied so I love it all my heart”*	
Planting	Teaching and giving tips on how to plant and encouraging people to grow cannabis	Picture of kratom tree with phrase
		*“not so long to be professional (on planting)”*
		*“can be eaten soon”*
Law Issue	*“Let’s sign your name on this site* *xxx.com* *to support the removal of cannabis from the drug schedule. Tell your friends too!”*	None
	*“Cannabis has lots of benefits (kill cancer, anxiolytic effect) and is less addictive compared to alcohol and cigarettes. Why is it illegal whereas alcohol is harmful to health but legally sold? I don’t understand Thai law.”*	
Marketing	- Plants, seeds, leaves and equipment were marketed.	- Mostly sell kratom leaves.
	*“Add me (Line ID xxx) if you want to know where to get cannabis seed.”*	*“If you need a large amount of kratom, please contact us. We sell them in limited quantities.”*
	*“We invite you to our page HighBong. We sell glass vaporizer and all kind of equipment for cannabis users. We also have a new page for gaining more customers.*	*“A large number of kratom leaves sold here. 1 kg 900 baht. 2 kg 850 baht. 3 and above 800/kg. Limited supply, please order now.”*
	*There may be only a few items but we plan to have more soon and ensure to you that we will send them right to your hand. We can guarantee delivery!”*	*“Only two months to grow. If you are interested, please leave your Line ID, we will contact you.”*
	- Link to webpage about the cannabis was embedded in some page.	
	- Pictures of products and packages and reviews from customers about the products they bought	
Pictures with or without text but Showing Usage	Picture of cannabis leaves and plants together with vapor or “bong” in different designs, and/or people (adult or child) who were smoking weed	Picture of kratom tea in the pot or glass which was ready to drink and/or with people drinking that tea near the pot
		*“In this hot weather, we need some sweet.” (with picture of kratom tea and a bottle of cough suppressant)*
Posts with Neutral tone		
Changing page cover or page profile (which we automatically categorized as neutral tone)	Picture of the plant or leaves	Picture of the plant or leaves
		Posting about weapons (Guns)
		Picture of smiling person
Sharing news about law or research finding without opinion	-News about seminar of the committee from the law reform commission of Thailand about the drug policy	None
	-News about HEMPLAND – the exhibition talking about present and future of cannabis industry of Thai	
	-News about decriminalization of drug use and possession for personal consumption from UNODC	
	-News about the future policy about cannabis tax payment to support education	
General greeting by administrator of any unrelated topic to the drug	-Picture or text related to king RAMA9 death	-Picture or text related to king RAMA9 death
	-Sharing video about Thai dancing	-Picture of brown color drink in soft drink bottle
	-Picture of a lighter with phase “one of the most common lost items”	-Updating page status by administrator *“Please add our new page. This page will be close out soon.”*
	-Updating page status by administrator *“I’m back. From tomorrow, I will update the page daily. Will get you some VDO! Have a good night everybody!!!”*	

This table describes posts with positive and neutral tone categorized by topic. The contents in cannabis page were slightly different from kratom page

Both drugs had deactivated pages, but new pages were found after 1 year. There was some weak evidence that the number of deactivated pages was higher for cannabis than kratom (*p* = 0.10). However, ten new Facebook pages for cannabis were found after 1 year, while only three new pages were found for kratom. During the entire study period, we evaluated a total of 24 cannabis pages (267 posts) and 15 kratom pages (206 posts).

### Online information for Cannabis on Facebook

The numbers of followers ranged from 225 to 208,695 for the twelve cannabis pages initially found. The average duration between the last ten posts was 46 days (SD 45.8, median 27 days, range 7–148 days). The tone of the posts was likely to be positive towards the use of cannabis (Table 2). Some posts were neutral, but a negative tone was not found. The total number of posts counted for some pages did not reach ten during the searching period. There was one other drug mentioned on the cannabis pages. The administrators mostly discussed cannabis, but one page also mentioned Procodyl® (promethazine hydrochloride). The tones of the posts, numbers of followers, and names of other drugs mentioned on pages for cannabis are shown in Table 3.

**Table 3:** Cannabis Facebook Pages in 2015 and 2016

Page	2015							2016						
	Followers in 2015	Tones (Number)			10 Posts Duration (days)	Other Drug(s) Mentioned on Page		Followers in 2016	Tones (Number)			10 posts Duration (days)	Other Drug(s) Mentioned on Page	
		P	N	U		Number	Name(s)		P	N	U		Number	Name(s)
CF1^a^	208,695	5	–	5	148	1	Procodyl	*Not available*	–	–	–	–	–	–
CF2	203,564	7	–	3	17	None	–	*Not available*	–	–	–	–	–	–
CF3	190,580	3	–	7	7	None	–	193,647	4	–	6	8	1	Kratom
CF4^b^	52,647	2	–	8	53	None	–	*Not available*	–	–	–	–	–	–
CF5	4356	9	–	1	7	None	–	*Not available*	–	–	–	–	–	–
CF6	3673	5	–	5	14	None	–	*Not available*	–	–	–	–	–	–
CF7	3267	4	–	6	41	None	–	3537	10	–	–	160	None	–
CF8	1956	10	–	–	10	None	–	1958	10	–	–	10	None	–
CF9	1262	9	–	1	18	None	–	*Not available*	–	–	–	–	–	–
CF10	897	4	–	6	93	None	–	*Not available*	–	–	–	–	–	–
CF11	247	1	–	9	36	None	–	*Not available*	–	–	–	–	–	–
CF12	225	3	–	7	104	None	–	*Not available*	–	–	–	–	–	–
CF13	–	–	–	–	–	–	–	389,902	10	–	–	8	None	–
CF14	–	–	–	–	–	–	–	100,699	7	–	3	3	None	–
CF15^a^	–	–	–	–	–	–	–	40,837	5	–	5	4	None	–
CF16	–	–	–	–	–	–	–	25,444	6	–	4	7	None	–
CF17^b^	–	–	–	–	–	–	–	7943	6	–	1	1^c^	None	–
CF18	–	–	–	–	–	–	–	7139	8	–	2	154	None	–
CF19	–	–	–	–	–	–	–	5885	10	–	–	3	None	–
CF20	–	–	–	–	–	–	–	2712	5	–	5	19	1	Kratom
CF21	–	–	–	–	–	–	–	1183	6	–	4	23	2	Ya-ba, Kratom
CF22	–	–	–	–	–	–	–	357	7	–	3	23	None	–
CF23	–	–	–	–	–	–	–	145	9	–	1	6	None	–
CF24	–	–	–	–	–	–	–	75	8	–	2	6	None	–

Number of pages, number of followers, number of posts categorized by tones, page activity and other drug mentioned in page were defined for cannabis pages both found in 2015 (12 pages) and 2016 (15 pages), *P* positive, *N* negative, *U* neutral

^a^Same name but different URL

^b^Similar shop name “Bong party” at the end of the page title

^c^= less than ten posts

One year later, most cannabis pages were not available (9/12 pages), and twelve new pages were found with similar trends in the tone of the posts. The news about the medical benefit and legalization were shared. In 2016, two pages used similar names to those of two pages found in 2015 that were not available when searching by URL. The remaining pages had an increasing number of followers, and the other drug groups mentioned on the cannabis pages were different from those in 2015. We found three cannabis pages that mentioned kratom (not including the kratom pages) and one page about Ya-ba - a tablet containing methamphetamine and caffeine [29]. The duration between the last ten posts was shorter among the cannabis pages, with an average of 29 days (SD 52.4, median 8 days, range 1–160).

### Online information for Kratom on Facebook

Followers for the twelve pages ranged from 89 to 7336. The average duration of the last ten posts was 85 days (SD 64.5, median 111 days, range 0–163 days). The tone of the posts was similar to those on the cannabis pages, in that there was more content in positive tones about kratom use. Content about legal issues, which was found on the cannabis pages, was not found on the kratom pages (Table 2). More than half of the kratom pages mentioned other drugs, as shown in Table 4. Two pages mentioned cannabis but were not found when searching for cannabis. Most pages mentioned controlled prescription drugs with sedative effects. The most common one was cough syrup containing antihistamines (chlorpheniramine or diphenhydramine) and ammonium chloride, which was categorized by the Thai Food and Drug Administration as a dangerous drug. These included A-Chlordyl®, Cephendryl®, Bephendryl®, A-waryl®, Iwadil®, Inadril®, and I-22 syrup®.

**Table 4:** Kratom Facebook Pages in 2015 and 2016

Page	2015							2016						
	Followers in 2015	Tones (Number)			Ten Posts Duration (days)	Other Drug(s) Mentioned on Page		Followers in 2016	Tones (Number)			Ten Posts Duration (days)	Other Drug(s) Mentioned on Page	
		P	N	U		Number	Name(s)		P	N	U		Number	Name(s)
KF1	7336	7	–	3	97	2	alprazolam, a-chlordyl	*Not available*	–	–	–	–	–	–
KF2	4030	9	–	1	125	3	cephendryl, bephendryl, a-chlordyl	4350	9	–	1	70	2	a-chlordyl, cannabis
KF3	4017	9	–	1	133	1	cannabis	4150	9	–	1	213	None	–
KF4	3241	10	–	–	128	1	a-chlordyl	*Not available*	–	–	–	–	–	–
KF5	2569	10	–	–	163	5	cephendryl, bephendryl, a-chlordyl, i-22 syrup, cannabis	2886	10	–	–	31	None	–
KF6	492	9	–	1	153	1	a-waryl	666	6	–	4	258	None	–
KF7	377	4	–	6	142	None	–	*Not available*	–	–	–	–	–	–
KF8	192	7	–	3	44	None	–	394	8	–	2	40	2	a-chlodyl, iwadil syrup
KF9	180	1	–	9	14	5	alprazolam,a-chlordyl, benadril, iwadil, inadril	*Not available*	–	–	–	–	–	–
KF10	134	6	–	1	0^c^	None	–	156	6	–	1	0^c^	None	–
KF11	123	5	–	5	15	4	cephendryl, bephendryl, inadril, a-chlordyl	*Not available*	–	–	–	–	–	–
KF12	89	5	–	5	1	None	–	2068	5	–	5	1	None	–
KF13	–	–	–	–	–	–	–	435	2	–	8	308	4	procodyl, tramadol, a-chlodyl, B5
KF14	–	–	–	–	–	–	–	214	–	–	2	14^c^	1	alprazolam
KF15	–	–	–	–	–	–	–	260	10	–	–	69	None	–

Number of pages, number of followers, number of posts categorized by tones, page activity and other drug mentioned in page were defined for kratom pages both found in 2015 (12 pages) and 2016 (10 pages), *P* positive, *N* negative, *U* neutral, ^c^ = less than ten posts

Five out of twelve of kratom Facebook pages were not available after 1 year, and three new pages were found. The other drugs mentioned in the kratom pages and the tone of the posts were similar to those in 2015. The median duration between the last ten posts was shorter than that in 2015, but the mean was approximately 100 days (SD 114.7, median 54.5 days, range 0–308).

## Discussion

The possession or use of cannabis or kratom is illegal in Thailand. This metanarrative contextualizes the content analysis that was conducted for this study. The majority of public information on Facebook regarding these drugs is positive for the use of the drugs. The contents posted on Facebook pages for both drugs tended towards convincing people to use and buy the drugs online by using invitation text and photos, and this tendency continued at a one-year follow-up. No warning regarding the harm of these drugs was observed. Cannabis pages tended to be more popular and more active than kratom pages.

Cannabis pages were available on Facebook with a high number of followers. At the one-year follow-up, their contents were in the same tones, and the number of followers remained high even though some pages had been deactivated. The content was similar to that found in prior studies conducted in the US [2, 3, 27]. People expressed their good feeling about cannabis, telling other people that they wanted to use it, inviting others to try, mentioning the benefits of the drugs on relieving stress and its medical purposes, suggesting that it should be legalized, and comparing it to other legal drugs by noting its greater benefits and lower risks to health. Posts against cannabis use were comparatively infrequent in the previous study [3] while we found none in our study.

Kratom was similar to cannabis that the tones of the posts on Facebook were positive and neutral rather than negative in tone. The post contents were mainly about inviting people to try using it and to observe its benefits. However, kratom was less popular by posting rate and number of followers than cannabis was. This effect could result from the fact that kratom is mainly used in the southern part of Thailand, unlike cannabis, which is more common nationwide. Moreover, cannabis social trends in Thailand could be driven by the legalization of recreational cannabis in the US in the recent years. Thus, we observed posts about legalization on the cannabis pages unlike the kratom pages.

Both drugs had deactivated pages, but new pages were found after 1 year. There were a higher number of deactivated pages and new pages for cannabis compared to the number of pages found initially. One limitation of our study was being unable to know the reasons for page deactivation, i.e., whether it was government action or by intention of the administrators to hide from government investigation. This finding could imply that cannabis sellers could be more alert and aware of the action of legal authorities, due to the more serious penalties for cannabis than for kratom under Thai law. They may deactivate and open new pages to avoid this action, and sometimes a similar name might be used for the old page followers to recognize and trace the new page [6]. In contrast, kratom is less popular and carries less serious punishments for possession or distribution than cannabis. This reason may be why fewer new pages and fewer deactivated pages were observed when compared with cannabis pages.

We also observed that online content such as advertising or marketing could raise awareness among regulators. This finding indicates that Facebook may also act as a distributor of the drugs. However, it could be useful that contact details or addresses could help direct investigators of the administrators of these pages to a possible virtual market place for further evaluation. Furthermore, advertising content must be a greater concern. Subjective opinions and recommendations given by users about these drugs without any scientific evidence to support them should be highlighted. In this study, we did not differentiate Facebook pages into user-generated or seller-generated pages, as this difference was not part of our initial aims in the study. However, similar content could be posted by both types of pages and be dangerous to customers if they believe it uncritically. Thus, media literacy education may be helpful, as also suggested in a prior study, which found that most media messages from retailers emphasize unproven health benefits without describing harm [1].

The trend of recommending other drugs to be used with cannabis was different from that of kratom. Cannabis pages rarely mentioned other drugs, which could indicate that it is primarily used alone. In contrast, kratom tended to be used with additional compounds to enhance the psychoactive effect by adding drugs and/or medication with a hypnotic effect. In Thailand, these drugs are categorized as dangerous drugs, controlled drugs, or illicit drugs that could be harmful to the user [16, 26]. Accordingly, the online information that encourages people to use a combination of these drugs could lead to more troublesome health outcomes.

At the one-year follow-up, increased page activity was observed for both drugs, and the posts were positive towards the use of the drugs. No postings educated people regarding the pros and cons of the drugs without suggesting opinions. Increasing the number of pages containing knowledge about the drugs or support systems for substance-using individuals, such as online therapy, maybe useful in order to facilitate accessibility for at-risk populations [14].

The strength of our study was that our searching was conducted on search engines that provided additional keywords that the general public could reach by a simple search. In addition, the one-year follow-up with the recorded URLs for old pages helped determine the activity of the pages and compared the trends of posts and tones changes within each page. New searches with the same keywords in the next year helped compare the page activities and the changes in post trends. Although new slang words might be missed that originated in the succeeding year, we hypothesized that only a few new slang words would be created within 1 year. Therefore, we believed that our initial keywords could cover most of the slang used among Thai people regarding these drugs.

The limitations of our study were that only Thai-language Facebook pages were searched, which would underestimate the number of postings with a hashtag that could be searchable through Facebook or pages with other languages which could also be accessed. Our study did not examine online information from other sites which may contain other educational information which could reflect other sources of the information that reaches the general population regarding these drugs.

## Conclusion

The current situation on Facebook regarding cannabis and kratom shows a somewhat positive attitude towards the use of these drugs. In addition, the content of some pages was trying to support legalization. No information about harm from these drugs was found through our search.
